# Supplementary material for: Microbial diversity patterns in the root zone of two Meconopsis plants on the Qinghai-Tibet Plateau
Source: PeerJ. 2023 May 24;11:e15361. doi: 10.7717/peerj.15361 (PMC10224674; doi:10.7717/peerj.15361)
Supplement: Supplemental Information 1 [file peerj-11-15361-s001.pdf]

Dear Academic Editor,

We commissioned a third party testing company to determine the soil elements. The original monitoring report is in in the attached.

Table 1 Soil testing basis and use of instruments

Test items	Detection method and standard number	Method Detection Limit,MDL	Testing instruments	Instrument number
Total nitrogen	the modified Kjeldahl Method (Chinese standard method HJ717-2014)	48mg/kg	Acid burette	3-DD50-01
Available phosphorous	the ascorbic acid colorimetric method (Chinese standard method HJ704–2014)	0.5mg/kg	Ultraviolet-visible Spectrophotometer UV1800PC	YQ-092
Available K	method NY/T889-2004	5mg/kg	AA-7020Atomic Absorption Spectroscopy	YQ-001
Organic matter	NY/T1121.6-2006 method	1.0g/kg	Acid burette	3-DD50-01
pH	pH metre	-	pH metre	Sartorius PB-10
Soil moisture	dying method	-	dying oven	-

Table 2 Summary of the soil properties of each sample

Site/*P* value	Sample	soil moisture (%)	Organic matter/ (mg/kg)	Available K/ (mg/kg)	Available P/ (mg/kg)	Total nitrogen /(mg/kg)	pH
M site	*M. horridula*	13.4	42.5	18	9.6	2430	8.13
M site	*M. integrifolia*	20.9	37.6	16	1.1	202	6.24
M site	Bulk soil	36.4	112	14	1.3	1750	7.14
D site	*M. horridula*	27.7	66.2	14	1.5	2880	6.94
D site	*M. integrifolia*	19.4	43.7	2	1.4	3370	7.21
D site	Bulk soil	6.5	72.6	14	4.6	3460	7.45
