# Supplementary material for: Microbial diversity patterns in the root zone of two Meconopsis plants on the Qinghai-Tibet Plateau
Source: PeerJ. 2023 May 24;11:e15361. doi: 10.7717/peerj.15361 (PMC10224674; doi:10.7717/peerj.15361)
Supplement: Supplemental Information 2 [file peerj-11-15361-s002.pdf]

报告编号：TBT-[2021]-0156 号

CMA
162612050010

正本

检 测 报 告

项 目 名 称： 6 个土壤样品理化性质的测定

检 测 类 型： 土壤

委 托 单 位： 西藏大学理学院

委托单位地址： /

编制人：[signature] 审核人：[signature] 批准人：[signature]

签发日期：2021 年 05 月 20 日

西藏博源环境检测有限公司

西藏博源环境检测有限公司 检验检测报告专用章

检测报告说明及声明

一、报告无“检测报告专用章”、“正本”章和骑缝章无效。

二、报告内容涂改增删无效；无编制、审核和批准人签字无效。

三、委托单位对本检测报告如有异议，请于收到报告之日(以邮戳为准或签收日)起七天内向本公司提出，逾期不予受理。无法保存、复现的样品，不受理申诉。

四、系委托方自行送检的样品，本公司只对当次样品检测数据负责，不对其来源负责；系受委托方委托，由检测方负责采样分析的样品，仅对当次检测有效。

五、未经本公司书面批准，不得复制（全文复制除外）检测报告。

六、未经本公司书面批准，本报告及数据不得用于商业宣传及其它非研究类用途，违者必究。

七、无资质认定（CMA）标志的报告和报告中标明不在资质认定（CMA）范围内的检查项目，其结果仅供委托方内部使用，不具有社会证明作用。

八、报告中的结果与特定的时间、特定的方法、特定的适用标准及所检样品有关，当采用不同的方法和标准对样品进行检测有可能得出不同的结果。

本公司通讯资料

联系电话：0891 6143625

传　　真：0891 6143625

E-mail：tibetbeyondtesting@163.com

邮政编码：850000

地　　址：拉萨市城关区蔡公堂路 109 号

一、项目概况

受西藏大学理学院委托，西藏博源环境检测有限公司对《6 个土壤样品理化性质测定》项目进行检测，于 2021 年 04 月 28 日至 05 月 17 日进行样品分析。

二、检测内容

2.1 土壤

2.1.1 样品名称

M1（MLS-1）、M2（MLS-2）、M3（MLS-3）、M4（DDC-1）、M5（DDC-2）、M6（DDC-3），共 6 个样品。

2.1.2 检测项目

全氮、有效磷、速效钾、有机质，共 4 个。

2.1.3 样品数量

共 6 个。

三、样品状态

样品编号	样品类型	样品状态
210156A101	土壤	棕黄色固体。
210156B101	土壤	棕黄色固体。
210156C101	土壤	黑色固体。
210156D101	土壤	褐色固体。
210156E101	土壤	黑色固体。
210156F101	土壤	褐色固体。

四、检测人员

贾正灿、徐仁茂、拉姆曲措。

五、质量控制和质量保证

5.1 质量控制措施

按照国家环境保护总局颁布的《环境监测质量保证管理规定（暂行）》要求，对布点、采样、分析测定、数据处理全程序进行质量控制。

（1）采样人员严格遵守采样操作规程，认真填写采样记录，按规定保存和运输样品；选择部分项目加采现场空白，每批样品按 10%加采平行样。

（2）监测分析方法采用国家颁布的标准分析方法或推荐方法，监测人员持有上岗证，所有监测仪器、量具均经过计量部门检定合格，并在有效期限内。

（3）水样测定过程中按规定进行质控样、平行空白、平行样测定。

（4）原始数据的填报、监测报告严格实行三级审核制度。

六、检测依据及使用仪器

表 6-1 土壤检测依据及使用仪器

序号	检测项目	检测方法及标准号	方法检出限	检测仪器	仪器编号
1	全氮	土壤质量 全氮的测定 凯氏法<br>HJ 717-2014	48mg/kg	酸式滴定管	3-DD50-01
2	有效磷	土壤 有效磷的测定<br>碳酸氢钠浸提-钼锑抗分光光度法<br>HJ 704-2014	0.5mg/kg	UV1800PC<br>紫外可见分光光度计	YQ-092
3	速效钾	土壤速效钾和缓效钾含量的测定<br>NY/T 889-2004	5mg/kg	AA-7020<br>原子吸收分光光度法	YQ-001
4	有机质	土壤检测 第六部分：<br>土壤有机质的测定<br>NY/T 1121.6-2006	1.0g/kg	酸式滴定管	3-DD50-01

七、检测结果

表 7-1 土壤检测结果

样品名称		M1（MLS-1）	M2（MLS-2）	M3（MLS-3）	M4（DDC-1）	M5（DDC-2）	M6（DDC-3）
接样日期		2021.04.28	2021.04.28	2021.04.28	2021.04.28	2021.04.28	2021.04.28
样品编号		210156A101	210156B101	210156C101	210156D101	210156E101	210156F101
序号	检测项目	检测结果					
1	全氮（mg/kg）	2.43×10^3	202	1.75×10^3	2.88×10^3	3.37×10^3	3.46×10^3
2	有效磷(mg/kg）	9.6	1.1	1.3	1.5	1.4	4.6
3	速效钾（mg/kg）	18	16	14	14	2	14
4	有机质（g/kg）	42.5	37.6	112	66.2	43.7	72.6
备注：该批次样品为委托方送样，本公司只对样品检测结果负责，不对其来源负责。							

——以下无检测数据——
