# Supplementary material for: Microbial diversity patterns in the root zone of two Meconopsis plants on the Qinghai-Tibet Plateau
Source: PeerJ. 2023 May 24;11:e15361. doi: 10.7717/peerj.15361 (PMC10224674; doi:10.7717/peerj.15361)
Supplement: Supplemental Information 3 [file peerj-11-15361-s003.docx]

**Test report (translation)**

Project Name: Determination of soil properties of 6 samples

Detection Type: soil ­­­­­ ­

Entrusted by: College of Science, Tibet University

Address of the entrusted unit:/

Tibet BoYuan Environmental Testing Co., Ltd.

**1. Overview**

Entrusted by the School of Science of Tibet University, Tibet Boyuan Environmental Testing Co., Ltd. tested the project of "Determination of Physical and Chemical Properties of 6 Soil Samples", and conducted sample analysis from April 28 to May 17, 2021.

**2. Test content**

2.1 Soil

2.1.1 Sample Name

M1 (MLS-1), M2 (MLS-2), M3 (MLS-3), M4 (DDC-1), M5 (DDC-2), M6 (DDC-3), a total of 6 samples.

2.1.2 Inspection items

Total nitrogen, available phosphorus, available potassium, organic matter, a total of 4 items

2.1.3 sample quantities

6 sample in total.

**3.Sample status**

| Sample number | Sample type | Sample status |
| --- | --- | --- |
| 210156A101 | soil | Solid brownish yellow |
| 210156B101 | soil | Solid brownish yellow |
| 210156C101 | soil | Black solid |
| 210156D101 | soil | Solid brown color |
| 210156E101 | soil | Black solid |
| 210156F101 | soil | Solid brown color |

**4. Inspectors**

Jia Zhengcan, Xu Renmao, Ram Qutso

**5. Quality control and quality assurance**

In accordance with the requirements of the "Provisions on the Management of Environmental Monitoring Quality Assurance (Provisional)" promulgated by the State Environmental Protection Administration, quality control is carried out on the whole process of distribution, sampling, analysis and measurement, and data processing.

(1) Samplers strictly abide by the sampling operating procedures, carefully fill in the sampling records, and store and transport samples according to regulations; Select some items to add site blanks, and each batch of samples will be 10% plus parallel samples.

(2) The monitoring and analysis method adopts the standard analysis method or recommended method promulgated by the state, the monitoring personnel hold the post certificate, and all monitoring instruments and measuring tools have been verified and qualified by the measurement department and are within the validity period.

(3) Quality control samples, parallel blanks and parallel samples are determined according to regulations during the determination of water samples.

(4) The three-level review system is strictly implemented for filling in and monitoring reports of raw data.

**6. Testing basis and instrument table**

6-1 Soil testing basis and use of instruments

| **Test items** | **Detection method and standard number** | **Method Detection Limit,MDL** | **Testing instruments** | **Instrument number** |
| --- | --- | --- | --- | --- |
| Total nitrogen | the modified Kjeldahl Method (Chinese standard method HJ717-2014) | 48mg/kg | Acid burette | 3-DD50-01 |
| Available phosphorous | the ascorbic acid colorimetric method (Chinese standard method HJ704–2014) | 0.5mg/kg | Ultraviolet-visible Spectrophotometer UV1800PC | YQ-092 |
| Available K | method NY/T889-2004 | 5mg/kg | AA-7020Atomic Absorption Spectroscopy | YQ-001 |
| Organic matter | NY/T1121.6-2006 method | 1.0g/kg | Acid burette | 3-DD50-01 |
| pH | pH metre | - | pH metre | Sartorius PB-10 |
| Soil moisture | dying method | - | dying oven | - |

7. Test results

| Sample name | M1(MLS-1) | M2 (MLS-2) | M3 (MLS-3) | M4(DDC-1) | M5(DDC-2) | M6(DDC-3) |
| --- | --- | --- | --- | --- | --- | --- |
| Date | 2021.04.28 | 2021.04.28 | 2021.04.28 | 2021.04.28 | 2021.04.28 | 2021.04.28 |
| Sample number | 210156A101 | 210156B101 | 210156C101 | 210156D101 | 210156E101 | 210156F101 |
| Detect items | Results | | | | | |
| Total nitrogen /(mg/kg) | 2.43x103 | 202 | 1.75x 103 | 2.88x103 | 3.37x103 | 3.46x 103 |
| Available P/(mg/kg) | 9.6 | 1.1 | 1.3 | 1.5 | 1.4 | 4.6 |
| Available K/(mg/kg) | 18 | 16 | 14 | 14 | 2 | 14 |
| Organic matter/(mg/kg) | 42.5 | 37.6 | 112 | 66.2 | 43.7 | 72.6 |
